# Electric Fields and Inflammation: May the Force be with You

**DOI:** 10.1100/tsw.2008.158

**Published:** 2008-12-25

**Authors:** Simon B. Brown, Ian Dransfield

**Affiliations:** MRC Centre for Inflammation Research, Queen's Medical Research Institute, University of Edinburgh, 47 Little France Crescent, Edinburgh, EH16 4TJ, UK

**Keywords:** membrane potential, electric fields, PECAM-1, integrins, galvanotaxis, wound healing

## Abstract

Integrins are a family of ubiquitous cell surface receptors comprising heterodimers of β and α chains that are required for cell adhesion and motility. Integrin-dependent adhesion and signaling is associated with major conformational changes in the ectodomain as it shifts from a low-affinity “bent” to a high-affinity “extended” structure. The ability of a cell to regulate dynamically the affinity or activation state of an integrin, and hence its binding to extracellular matrix or cell adhesion molecules, is assumed to be driven by intracellular signaling events transmitted by protein binding to the cytoplasmic tail. The binding of an integrin to its ligand can then transmit signals back into the cell to regulate the formation of a macromolecular focal adhesion complex that effectively anchors the cytoskeleton to the adhesion site. Many proteins have been reported to associate physically and functionally with integrins, leading to altered signaling events. A particularly intriguing molecular association exists between integrins and transmembrane proteins that gate the movement of charge, especially voltage-gated potassium channels, although the significance of this interaction is not understood. Although ample evidence indicates that the engagement of integrins can promote potassium efflux by both excitable and nonexcitable cells, we speculate the converse, that the activation state of integrins is dynamically regulated by changes in a transmembrane potential. In this way, direct-current electric fields generated at a site of tissue injury can promote the galvanotaxis or directed migration of cells involved in tissue repair and inflammation.

## THE PLASMA MEMBRANE AS A MOLECULAR AND IONIC SELECTIVITY FILTER

As inflammatory cell biologists, we readily appreciate that the innate and adaptive immune response of multicellular organisms to tissue injury and infection is a beneficial host response that is driven by a diverse array of receptor-mediated events. Specifically, a "lag-free" inflammatory response by the afflicted stroma and infiltrating immune cells depends on the integration of multiple soluble ligand and cell-pathogen, cell-matrix, and cell-cell binding events[1]. To put it another way, we view the plasma membrane and the receptors it expresses as a molecular selectivity filter in determining how a cell responds to its external environment[2].

The plasma membrane, however, is also a selectivity filter, or permeability barrier, for the free movement of ions, which can be used to store cellular energy and generate transmembrane voltage potentials. It can therefore come as no surprise, given that all molecular interactions are electrical in nature, that direct-current electrical fields (dcEF) generated by cells and tissues can have a profound effect on cell behavior[3,4,5]. This is perhaps best appreciated by modern-day electrophysiologists working with the action potentials of neuronal cells and the fast electrical conductance changes associated with musculoskeletal contraction. In contrast, the longer-lasting dcEF effects observed in wound healing, directed cell migration, and transcriptional regulation are less well known or understood. This review will focus on how endogenously derived dcEFs can influence both leukocyte migration and adhesion within the context of an inflammatory response. This review is prompted by our desire to understand why integrins can both physically and functionally associate with voltage-gated ion channels (Table 1, Parts A and B)[5]. Although integrin engagement is known to activate voltage-gated ion channels, we will argue that this coupling is two-way, and that the electrical properties of cells critically influence integrin function.

## ENDOGENOUSLY DERIVED AND PHYSIOLOGICALLY RELEVANT ELECTRIC FIELDS

Before we discuss the effect of physiologically relevant dcEFs on cell behavior, we need to first describe the various electrostatic potentials that lipid bilayers and stratified tissue can give rise to: a translipid[1] potential ($\Phi m$), a surface potential ($\Phi s$), a dipole potential ($\Phi d$), and a transcellular potential[6,7,8,9]. In physical terms, a potential difference is simply the amount of work that is required to move a unit electric charge from one point to another against an electric field and is synonymous with voltage, which for cells and tissues is typically measured in millivolts (mV). An electric field is defined as the voltage difference per unit distance and is a vector quantity, i.e., it has both magnitude and direction. Cell biologists, however, are perhaps more familiar with a **membrane potential** ($\Delta V$), which is the voltage difference that exists across a plasma membrane between the extra- and intracellular bulk aqueous phases. Thus, a membrane potential represents the sum of the surface and translipid potentials, and is the only potential difference that is measured directly by patch-clamp techniques (Fig. 1).

### Membrane Potentials

A cell establishes a potential difference across a plasma membrane through the expression of a plethora of ion porter, antiporter, and pump exchangers that function to establish differences in the concentration of various ions across that membrane. The hydrophobic nature, or high electrical resistance, of a lipid bilayer ensures that the plasma membrane is effectively impermeable to the free diffusion of those ions down their concentration gradients. Free movement of ions occurs through ion-selective pores that can either be opened or closed in response to a variety of "gating" mechanisms, including post-transcriptional modification, ligand binding, or voltage gating. Voltage gating is achieved when an electric dipole found within the voltage sensor of a voltage-gated ion pore is electrically coupled to the translipid potential and, as such, probably represents our foremost appreciation for the effect of electric fields on molecular and cellular function.

[1] This is more commonly referred to as a transmembrane potential, but is often confused with $\Delta V$, the transmembrane potential that exists between the extra- and intracellular bulk aqueous phases.

**TABLE 1**
**β1 and αvβ3 Integrins Associate with Voltage-Gated $K^+$ Channels**

**Part A**

| K+ Channel | Integrin | Accessory Protein | Association | | Cell Type Studied | Assay | Ref. |
|---|---|---|---|---|---|---|---|
| A | B | C | A∩B | A∩C | | | |
| Kv1.3 | β1 | | P | | T-lymphocytes | Adhesion to fibronectin (Fn) | 35 |
| Kv1.3 | β1 | | P | | Melanoma | Readhesion/FRET | 50 |
| Kv1.3 | ? | | F | | Primary cortical microglia | Chemokinesis, chemotaxis, β1 integrin expression | 51 |
| Kv1.3 | ? | T-type $Ca_v$ | F | | Neutrophils | | 23 |
| Kv1.4 | | | | | MDCK-F | Migration | 52,53 |
| Kv2.1 | | FAK | | P | HEK293; CHO; MEF | Readhesion and scratch wound | 25 |
| | | | | | Primary cortical neurons and glia | Readhesion and migration | |
| Kv11.1 | β1 | VEGF-R's | P | | Leukemic cells | Chemokinesis | 54 |
| Kv11.1 | α5β1 | PECAM-1 | F | P | Macrophages, erythroleukemic cell | Binding of Fn-coated latex beads and apoptotic cells | 34 |
| Kv11.1 | β1 | G protein | P | | Neuroblastoma | Readhesion, binding of Fn-coated latex beads and neurite outgrowth | 55,56 |
| Kv11.1 | β1 | FAK | P | | Neurroblastoma, HEK-hERG1 transfectants | Readhesion on Fn, FAK activation, co-IP studies | 26 |
| Kv11.1 | β1 | G protein | P | | Preosteoclastic leukemic cell | Adhesion, osteoclast formation, αvβ3 expression | 57 |
| $BK_{Ca}$ | αvβ3 | | F | | Endothelial cells | Effect of Vn on $[Ca^{2+}]_i$ | 58 |
| $BK_{Ca}$ | α5β1 | FAK, L-type $Ca_v$ | F | | Arteriolar myocytes | Fn-induced current (patch-clamp) | 27 |
| $BK_{Ca}$ | α5β1 | | | | Erythroleukemic cells | Binding of Fn-coated latex beads and Fn-induced hyperpolarization | 59 |
| Kir3.1/Kir3.4 | β1 | | P | | Xenopus oocytes; CHO | ACh-evoked currents, co-IP | 60 |
| Kir4.1 | α9β1 | | F | | MEF; CHO | Scratch wound | 61 |
| $K_V$, $K_{ATP}$, $K_{IR}$ | αvβ3 | | F | | Cremaster arterioles | RGD-mediated vasodilation | 62 |
| | α4β1 | L-type $Ca_v$ | | F | Cremaster arterioles | Vasoconstriction and L-type $Ca^{2+}$ currents | 63 |
| $K_V$, $K_{IR}$ | α4β1 | SOCCs | | | THP-1 | $Ca^{2+}$-mediated monocyte function | 64 |

NB: Directed migration in response to a scratch wound is an electrotactic response.

Association between K+ channel and integrin (A∩B) or accessory protein (A∩C) is either physical (P) or functional (F).

**TABLE 1**
**β1 and αvβ3 Integrins Associate with Voltage-Gated $K^+$ Channels**

**Part B**

| K+ Channel A | Subcellular Localization | Integrin Ligation | Biological Effect of K+ Efflux | Biological Effect of Raising $[K^+]_o$ | Biological Effect of $K^+$ Channel Blockers | Ref. |
|---|---|---|---|---|---|---|
| Kv1.3 | n.d. | n.d. | Adhesion | Promotes adhesion, chemokinesis, and β1 integrin activation | Blocks adhesion | 35 |
| Kv1.3 | Uniform distribution on resting cells | Promotes complex formation | Adhesion | n.d. | Blocks complex formation | 50 |
| Kv1.3 | n.d. | Supports migration | Migration | n.d. | Blocks migration | 51 |
| Kv1.3 | Clusters at leading edge | | | | Blocks dcEF detection | 23 |
| Kv1.4 | Clusters at leading edge | n.d. | Migration | n.d. | n.d. | 52,53 |
| Kv2.1 | Clusters at leading edge | Fn hyperpolarizes | Migration | Blocks migration | Blocks migration | 25 |
| Kv11.1 | n.d. | TS2/16 hyperpolarizes | Migration | n.d. | Blocks VEGF-R signaling and migration | 54 |
| Kv11.1 | Uniform distribution on resting cells | n.d. | Poor binding | Promotes adhesion | Promotes adhesion | 34 |
| Kv11.1 | n.d. | Fn and Vn hyperpolarize | Neurite outgrowth and FAK phosphorylation | n.d. | Blocks neurite outgrowth and FAK phosphorylation | 55,56 |
| Kv11.1 | Uniform distribution on resting cells | Fn hyperpolarizes | FAK phosphorylation | n.d. | Inhibits hERG/ FAK complex formation | 26 |
| Kv11.1 | n.d. | Fn and anti-β1 hyperpolarizes | Differentiation | n.d. | Impairs αvβ3 increased expression | 57 |
| $BK_{Ca}$ | n.d. | Increased $[Ca^{2+}]_i$ | Increased $[Ca^{2+}]_i$ | n.d. | Blocks rises in $[Ca^{2+}]_i$ | 58 |
| $BK_{Ca}$ | n.d. | Fn hyperpolarizes | Vascular tone | n.d. | Blocks constriction | 27 |
| $BK_{Ca}$ | n.d. | Fn hyperpolarizes | n.d. | n.d. | n.d. | 59 |
| Kir3.1/Kir3.4 | n.d. | Not tested directly | Putative neurosecretion | ACh densensitizaton | n.d. | 60 |
| Kir4.1 | Leading edge | Fn and Tn hyperpolarize | Migration | n.d. | Blocks migration | 61 |
| $K_V$, $K_{ATP}$, $K_{IR}$ | | Hyperpolarizes | Reduced $[Ca^{2+}]_i$ | Blocks vasodilation | Blocks RGD-induced vasodilation | 62 |
| | n.d. | LDV increased $[Ca^{2+}]_i$ | n.d. | n.d. | n.d. | 63 |
| $K_V$, $K_{IR}$ | mAb-mediated clustering | Increased $[Ca^{2+}]_i$ | Increased $[Ca^{2+}]_i$ | Blocks $[Ca^{2+}]_i$ | Blocks $[Ca^{2+}]_i$ | 64 |

n.d., not determined.

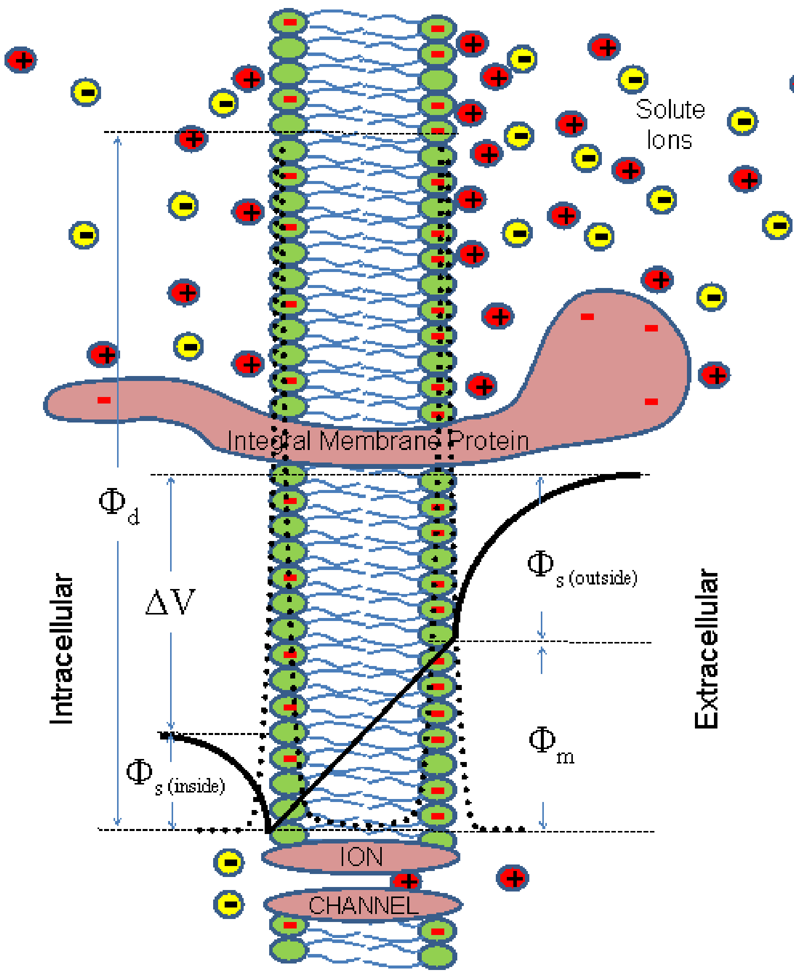

**FIGURE** 1. Schematic diagram representing the spatial distribution of the electric potential profile across a plasma membrane. Cross-section of a plasma membrane showing the voltage relationship between surface (Φs) and translipid (Φm) potentials with respect to a membrane potential difference (ΔV) across the membrane between the extra- and intracellular bulk aqueous phases. Φm is more commonly known as the true transmembrane potential and is the potential that affects voltage-sensitive membrane proteins, especially those that gate the movement of charge. To obviate any confusion between Φm and ΔV as transmembrane potentials, we will refer to Φm as a translipid potential. Also shown is the electric profile for a dipole potential due to the tight packing and alignment of electrical dipoles within the headgroups of lipids. The magnitude of Φs and its ability to extend into the bulk aqueous phase is determined by the density of surface charge and the concentration of cations within the bulk aqueous phase, which serves to screen surface charge. ΔV is determined by the permeability of various ions as they diffuse down their concentration gradients.

Under "resting" conditions, the membrane potential (ΔVrest) of all cells is determined predominantly by $K^+$ permeability[9]. Thus, in most cases, we are concerned with chemical gradients established by the Na/K-ATPase exchanger that concentrates $K^+$ within a cell (typically 155 m*M*) while pumping $Na^+$ out (typically 12 m*M*) in which the extracellular concentration for each ion is typically 4 and 145 m*M*, respectively. Application of the Nernst equation now allows us to define an equilibrium or reversal potential for $K^+$ (approximately –90 mV) and $Na^+$ (approximately +67mV) as the membrane potential at which the voltage force will exactly balance the chemical force for the free movement of ions down their concentration gradient. That is, as an ion diffuses through an open, but selective, ion pore, it will leave behind a counter ion that gives rise to a separation of charge across that membrane, which increases to the point where a potential difference, an **equilibrium potential**, resists further ion diffusion and the resultant nett current falls to zero. Summing over all ions, a resting membrane potential therefore represents the balance of competing equilibrium potentials that arise from the various membrane permeabilities that predominate when a cell is at rest. In resting cells, a more negative resting membrane potential reflects greater $K^+$ permeability, which serves to pull ΔVrest towards the equilibrium potential for $K^+$. Finally, the difference between an actual and equilibrium membrane potential represents the electrical driving force that is available to move an ion across that membrane.

## Transcellular Potentials

The spatial and temporal separation of various ion pumps and porter systems within a membrane can also result in polarized differences in ion concentrations within and without a cell (Fig. 2a). This is most commonly achieved in tissues and organs that are surrounded by a layer of epithelial cells where the $Na^+$/$K^+$ ATPase pump is localized to the basolateral surface, while $Na^+$ pores are found within the apical face, resulting in the nett movement of $Na^+$ from the bulk extracellular fluid at the apical to basolateral surface[10,11,12]. Tight junctions between contiguous cells can then provide high electrical resistance that prevents leakage of ions down their concentration gradients between cells (paracellular conductance). When coupled to $Cl^-$ movement in the opposite direction, as occurs in the epithelium of the lung, this can then give rise to transcellular potential differences of tens of millivolts and dcEFs on the order of 50–500 mV/mm[3,13]. Thus, not only will there be differences in the membrane potentials at the basolateral and apical surfaces, but there will also be an electric field gradient within the cell that will have significant polarizing effects on intracellular ion gradients and molecular organization.

## Surface Potentials

**Surface potential** is the potential difference between the negatively charged membrane surface and the bulk aqueous phase. It is dependent on the density of cell surface–charged molecules, including protein and lipid head groups, and arises from an incomplete quenching of the nett excess negative electric charge[4]. It is important to note that ΔVrest is essentially independent of changes in surface potential, since ΔVrest must still balance the intra- and extracellular concentrations and membrane permeabilities of the various ions. Therefore, a surface potential merely serves to reduce the potential difference Φm between the inner and outer surface of the membrane without affecting ΔVrest (Fig. 1). On the other hand, the nature and concentration of the ions in the bulk extracellular fluid surrounding a membrane will have a profound effect on Φs due to ion shielding effects[14]. For the purpose of this review, our interest in surface potential only extends so far as it contributes to a membrane potential and the movement of ions (i.e., current), even though surface potential has an important bearing on determining the structure of proteins and the binding of charged molecules, including proteins, to either the membrane or membrane-bound receptors.

## Dipole Potentials

The fourth electrostatic field associated with a lipid bilayer is the **dipole potential,** which originates from the dipolar structure of the head groups in lipid molecules that align themselves at the lipid-water interface. This has been estimated to be as high as 300–400 mV, but will decay to 2 mV over a small distance of 8 Å or about three water molecules. In contrast, the influence of a surface potential will extend as far as 3–5 nm for a pure phospholipid bilayer, and is significant when compared to the size of proteins and the thickness of a protein glycocalyx. Although Φd is thought to be important for the behavior of peptides or proteins within

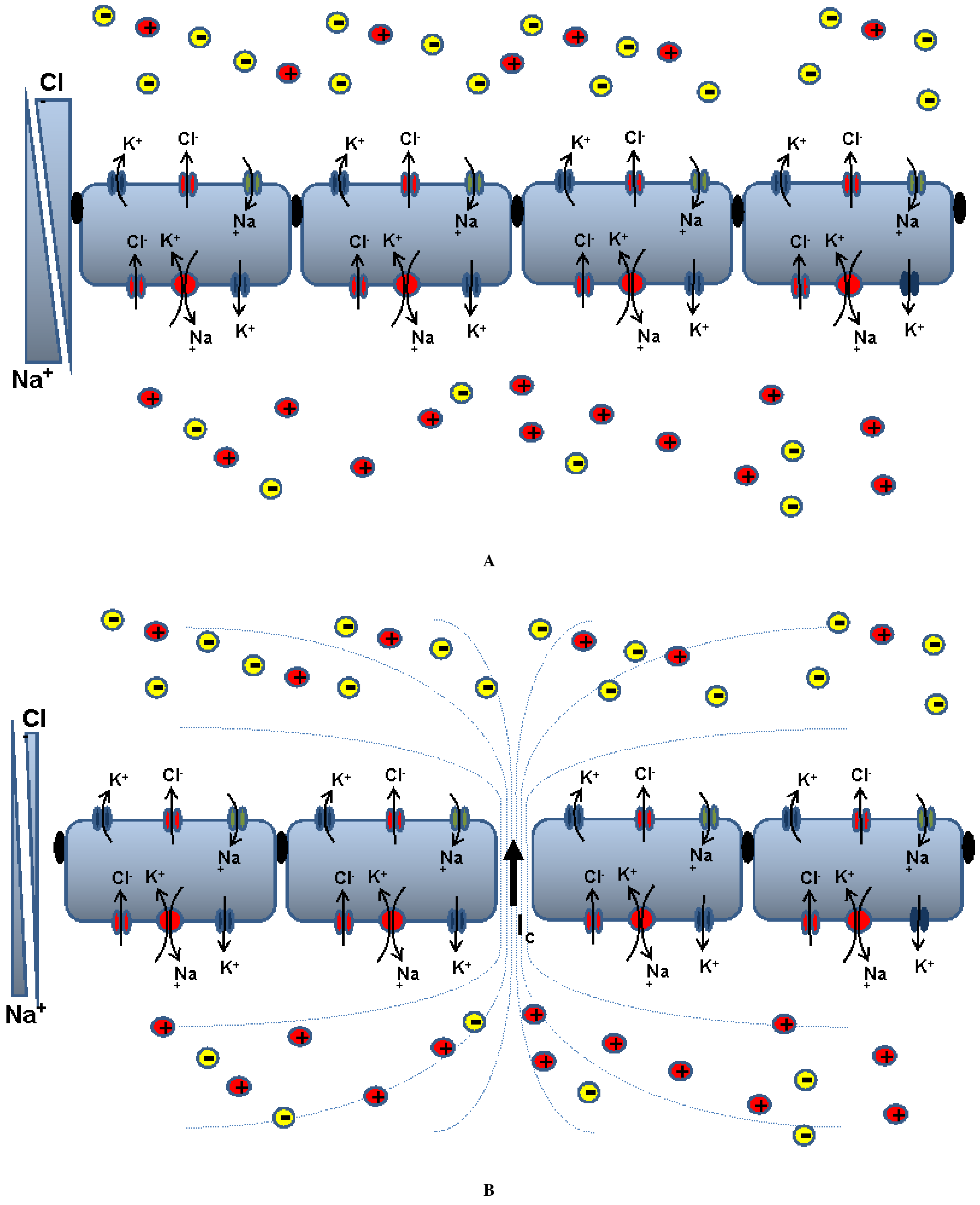

**FIGURE 2.** The origins of a transcellular potential. (A) A transcellular potential is established when ion transport across a contiguous layer of cells is selective and directional, resulting in ion concentration gradients between the bulk aqueous phase at the apical and basolateral surfaces. High electrical resistance that impedes paracellular conductance is maintained by tight junctions between adjacent cells in undamaged tissue. (B) Following tissue injury or a breakdown in the tight junctional complexes, a short circuiting of the voltage difference occurs and electric field vectors will be generated lateral to the plane of the monolayer as current ($I_c$) flows from areas of high resistivity to low. By convention, current flows in the direction of positive charge. The potential difference across the blood-brain barrier has been measured *in vivo* at 3–4 mV, suggesting direct-current electric fields as high as 100–200 mV/mm across the endothelial barrier, which is more than sufficient to promote the galvanotaxis of leukocytes.

cholesterol-rich lipid rafts, the effect on cellular physiology is not well understood and will not be discussed further[8].

We are now in a position to discuss the effect of electric fields on cell behavior.

## THE SHORT CIRCUITING OF MEMBRANE POTENTIALS IN INJURED CELLS AND TISSUES

As stated by Carl Nathan, "when injury and infection coincide, the goal (of any host inflammatory response) is to react as quickly as possible to terminate the spread of infection, even at the cost of further tissue damage"[1]. The simplest injury that we can consider is a cut to the skin where, as long ago as 1843, it was shown that ~1 µA of current can be detected[3]. This current represented what was already known on a single cell basis as an "injury" potential, a direct-current voltage difference due to the short circuiting of a membrane potential[3,4,14]. In a multicellular organ such as the skin, this injury potential represented the short circuiting of a transcellular (or transepithelial) potential that generated electric field vectors lateral to the plane of the epithelium as current flowed from areas of high resistivity (intact tight junctions) to the cut, where resistivity was low (Figs. 2b and 3). More recent studies have shown that the electric field caused by a cut to an epithelium will drop off exponentially back along the surface of the epithelium with a length constant of 330 µm, i.e., the distance back from the wound edge where the voltage gradient has decayed to 37% (1/e) of its maximum value[3]. Thus, the short circuiting of transepithelial potentials can be sensed over 1–2 mm from the wound and will persist until repair and re-epithelialization is complete.

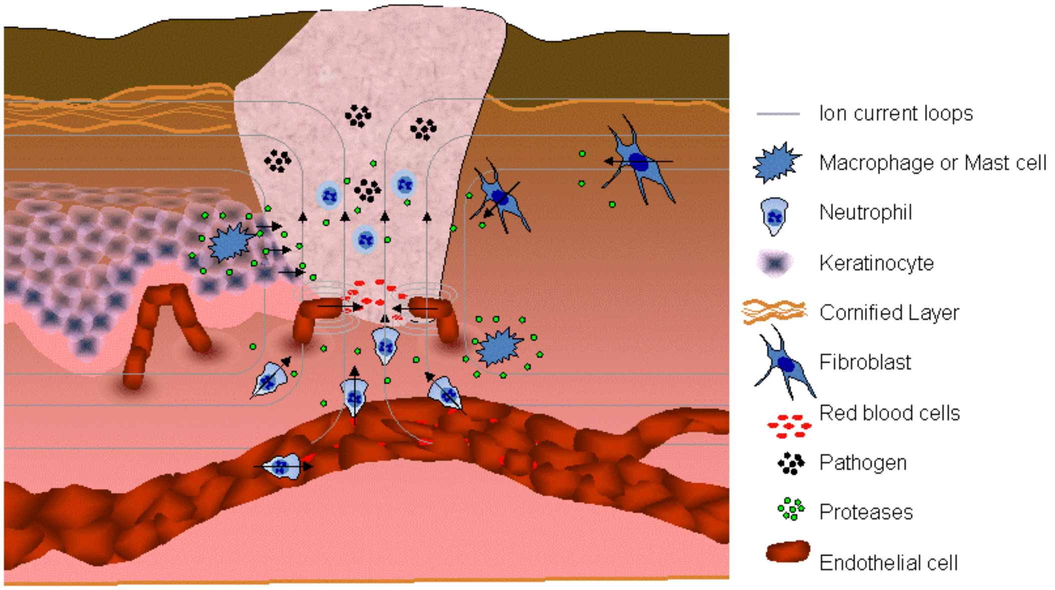

**FIGURE 3.** The role of electric fields in governing a host's response to tissue injury and infection. The short circuiting of transcellular potentials that comprise vascular capillary beds and epithelium help to recruit and guide neutrophils from the circulation towards the site of tissue damage. In addition, dcEFs will also promote the migration of stromal cells into the wound to bring about tissue repair where proteases not only function to break down physical barriers to cell migration, but also promote electrical conductance.

The principles of a transepithelial potential can easily be extended to an intact vascular capillary bed that comprises a monolayer of tightly apposed endothelial cells. Although transcellular potentials across the endothelial blood-brain barrier are small (~3–4 mV) when compared to a transepithelial potential, it is worthwhile bearing in mind that this is still sufficient to generate dcEFs on the order of 100–200 mV/mm. Furthermore, a vascular endothelium will associate with an epithelium where electric field vectors are additive[15]. A cardinal feature, and immediate early response, of inflammation is increased vascular permeability and edema, the leakage of water and plasma protein across the capillary wall that will undoubtedly result in increased ion conductance. Therefore, we can expect that an early response to tissue injury will be the short circuiting of a transendothelial potential in which underlying tissue damage will contribute to the generation of dcEFs.

## GALVANOTAXIS AND DIRECTED CELL MIGRATION

Galvanotaxis, or electrotaxis, is the directional movement of cells under the influence of an electric field, typically assessed by exposing cells *in vitro* to an applied external dcEF at physiologically relevant strengths[3,4,14,16]. It is important to state that not all cells will respond to an electric field by undergoing migration and for those that do, they will not all migrate in the same direction. Precisely why certain cells migrate to the cathode (negative pole) while others migrate to the anode (positive pole) is not known, even when cells are of the same lineage. Important for our discussion, however, is the finding that while epithelial cells, endothelial cells, and leukocytes are expected to migrate towards the cathode, i.e., into the wound, fibroblasts are expected to migrate from the opposite direction towards the anode to perhaps plug the gap (Fig. 3). These electrical cues are undoubtedly complemented by chemical cues that govern the chemotactic responses of inflammatory and stromal cells. Although dcEFs can establish chemical gradients, which might suggest that galvanotaxis is an epiphenomena, it is clear that galvanotaxis can occur both in the absence of and against a chemical gradient[3,17]. Thus, dcEFs may have a role in the directed migration and spatial organization of the inflammatory and wound healing response. Unfortunately, and to the best of our knowledge, studies to monitor the electrotactic responses of leukocytes to damaged tissue have not been reported.

## MOLECULAR COUPLING OF DCEFS TO DIRECTED CELL MIGRATION

Key to any cell movement is the acquisition of a polarized morphology, the asymmetric turnover of integrin-dependent focal adhesions and the dynamic rearrangement of a cortical cytoskeleton[18,19,20,21]. At its simplest, cell migration involves the elongation of actin filaments and formation of focal adhesions at the leading edge, disassembly of focal contacts at the trailing edge, and intracellular actomyosin-dependent contractility that propels the cell forward. Focal adhesions form when integrins bind extracellular matrix and bring together cytoskeletal and signaling proteins at the cytoplasmic face of the plasma membrane. Integrins are the conduit with which cells integrate extracellular "outside-in" and intracellular "inside-out" signaling events. Therefore, any mechanism that attempts to explain how cells can sense and convert electrical field signals into directed migration (independent of any chemotactic gradient) must impinge on these dynamic events, and presumably at the level of integrin function.

### Calcium Influx in Nonexcitable Cells

As indicated, physiological dcEF can be on the order of 500 mV/mm. For a neutrophil migrating on a surface or in a tissue (assuming it can extend up to 40 μm from the leading to trailing edges), there can exist as much as a 20 mV difference across the cell where neutrophils migrate towards the cathode. This has the effect of imposing a polarization on the intracellular cations toward the leading edge relative to the trailing edge, while reducing the membrane potential across the leading edge relative to the trailing edge (Fig. 4)[16]. In other words, a cell will be polarized with the leading edge depolarized relative to the

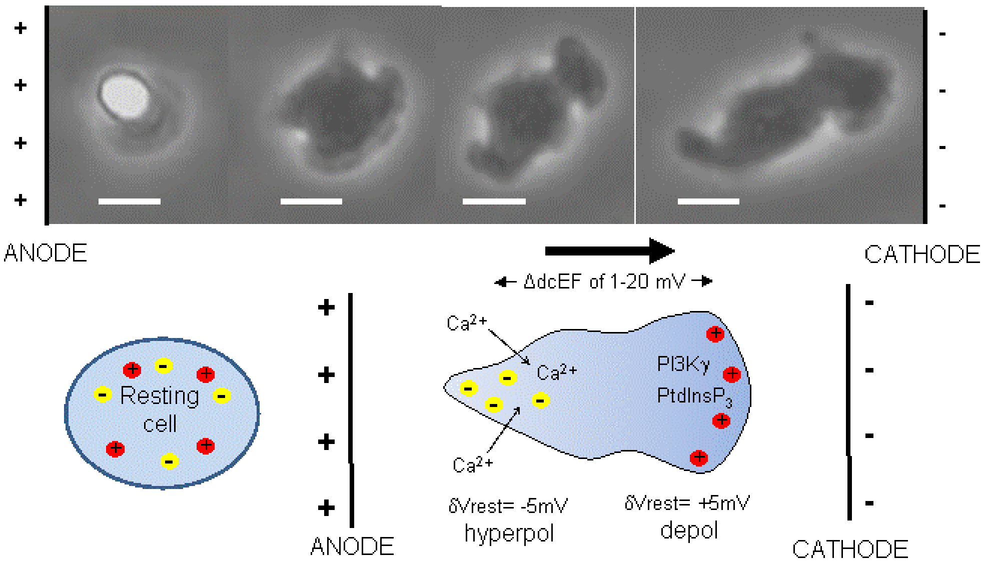

**FIGURE 4**. Electrical migration of cells. Neutrophils respond to an electric field by migrating towards the cathode (top panel). This serves to polarize ion solute species within cells by electrodiffusion. It has been estimated that dcEFs of 10–100 mV/mm will depolarize the leading "cathodal" edge by as much as 5 mV, whereas the trailing "anodal" edge will hyperpolarize by an equivalent amount. Such a difference in membrane potential will affect the driving force for calcium entry, which will be polarized towards the trailing edge and diffusing intracellularly towards the leading edge. In addition, PtdIns(3,4,5)$P_3$ is also known to polarize at the leading edge. We speculate that membrane depolarization at the leading edge "primes" integrins for binding extracellular matrix, while hyperpolarization can disrupt integrin-mediated adhesion in a galvanotactic neutrophil.

trailing edge (assuming the immediate bulk extracellular concentration of ions remains constant). Although controversial, it is generally assumed that neutrophils, like macrophages, are nonexcitable and do not express voltage-gated $Ca^{2+}$ channels[22,23]. This would imply that a difference in membrane potential across a cell cannot be responsible for preferentially activating a voltage-gated calcium channel at the leading edge to promote calcium entry. Rather, there would be a greater driving force for $Ca^{2+}$ entry (equilibrium potential ~120 mV) at the trailing edge relative to the leading edge, which may promote actomyosin contractability preferentially at the rear of the cell with expansion at the leading edge[16]. Although such a mechanism has not been tested or verified for leukocytes, dcEFs are known to induce intracellular calcium waves that emanate from the hyperpolarized side of a human prostate cancer cell line and fish keratocytes[reviewed in 16]. It is therefore of interest that calcium waves have also been observed in polarized neutrophils[24].

## Electrical Regulation of Focal Adhesion Kinase (FAK)

The stability of a focal adhesion, and hence a cell's propensity to migrate, is dependent on the phosphorylation state of nonreceptor focal adhesion tyrosine kinases, such as FAK or pyk2, that constitutively associate with the β-subunit of integrin receptors and serve as scaffolding proteins for the recruitment and activation of downstream signaling partners[21]. Of interest are reports that integrin-dependent binding of either CHO or SH-SY5Y neuroblastoma cells to fibronectin can promote FAK association with either Kv2.1 or Kv11.1 potassium channels[25,26,27]. Importantly, adhesion-dependent stimulation of $K^+$ efflux was essential for src-dependent phosphorylation of FAK at residue Y397 and perhaps Y576/Y577 (Table 1, Part B)[25]. Since phosphorylation of Y397 is especially important for the binding of several downstream signaling molecules, including the p85 subunit of PI-3K, voltage-gated potassium channels may provide a unique voltage-dependent mechanism that underlies FAK activation and directional migration. It is important to note, however, that $K^+$ efflux was secondary to integrin engagement.

## Electrical Regulation of Phosphatidylinositol-3,4,5-Trisphosphate

FAK is a promiscuous docking protein with respect to the signaling proteins that it can bind. FAK can therefore activate a number of intracellular signaling pathways, including those that regulate cell motility, division, and survival[21,28]. Our emphasis on PI-3K, however, is deliberate. A critical second messenger involved in regulating cytoskeletal rearrangements and cell adhesions other than calcium (discussed above) is a labile phosphatidylinositol-3,4,5-trisphosphate (PtdIns(3,4,5)P3) pool. The PtdIns(3,4,5)$P_3$ pool is regulated by the balance of competing lipid kinase and phosphatase activities; PI-3K generates PtdIns(3,4,5)$P_3$ while PTEN hydrolyzes PtdIns(3,4,5)$P_3$ to PtdIns(4,5)$P_2$. Both PtdIns(3,4,5)$P_3$ and PtdIns(4,5)$P_2$ are important second messengers in regulating cytoskeletal and lipid membrane dynamics[29,30,31]. In addition, PtdIns(4,5)$P_2$ is an activating ligand for many voltage-gated $K^+$ channels, including Kv11.1, suggesting a complex interplay between the PtdIns(3,4,5)$P_3$ pool, the cytoskeleton, and membrane potential[32]. It is also worth mentioning that the turnover of lipid phosphoinositides at the intracellular face of the plasma membrane has a profound affect on the inner surface potential and the recruitment of intracellular signaling molecules that are required for processes such as phagocytosis[33]. It is therefore significant that the intracellular level of PtdIns(3,4,5)$P_3$ is elevated at the leading migratory edge of cells undergoing galvanotaxis[17]. The importance of PtdIns(3,4,5)$P_3$ in mediating cellular responses to an external dcEF has been further established using cells that were deficient in either PTEN or p110γ, the catalytic subunit of a PI-3K expressed by leukocytes, where directed migration was either accentuated or attenuated, respectively[17].

## MEMBRANE DEPOLARIZATION AND INTEGRIN ACTIVATION

As already alluded to, cell adhesion and integrin engagement at the leading edge of a migrating cell can lead to FAK-dependent PI-3K activation and increased PtdIns(3,4,5)$P_3$ levels. This series of events, however, cannot explain satisfactorily how integrin activity in response to a dcEF was affected in the first place. It is possible that intracellular stores of integrins are mobilized preferentially to the leading edge and are therefore more likely to form new adhesions to drive $K^+$ efflux, FAK activation, and re-establish a ΔVrest. Unfortunately, we are unaware of any experimental evidence that might support the preferential recruitment of integrins to the leading edge. Nevertheless, it is established that integrin engagement either by cell binding to extracellular matrix and its components or by integrin-activating antibodies will activate $K^+$ efflux that can lead to cell hyperpolarization, adhesion, and spreading (Table 1, Part B). Cross-talk between $K^+$ channels and integrin function is further supported with the use of $K^+$ channel blockers that inhibit cell adhesion. It is notable, however, that the depolarization of lymphocyte and macrophage membrane potential can also result in the activation of β1 integrins[34,35]. Thus, depolarization might favor integrin-dependent binding events that, in turn, promote $K^+$ efflux and hyperpolarization, or repolarization when working with cells that have been removed from their substratum (Table 1, Part B). The question we wish to ask is whether membrane depolarization, which would occur at the leading edge of a migrating cell towards the cathode, or the binding of an apoptotic cell by a macrophage, can promote a conformational change within β1 integrins that is more permissive for ligand binding[34]. This was a question first raised by the seminal work of Levite et al.[35].

## EVIDENCE FOR MEMBRANE POTENTIAL MODULATION OF RECEPTOR AFFINITY

A precedent now exists by which membrane potential affects the affinity of m1 and m2 muscarinic receptors for acetylcholine[36]. These G-protein coupled receptors (GPCRs) were found to display charge-movement-associated currents analogous to voltage-gated ion channels that were responsible for affinity modulation. Interestingly, the galvanotaxis of keratinocytes is mediated by muscarinic receptors that are known to associate with β1 integrins and are regulated by integrin binding to ECM[37,38,39]. Muscarinic receptors are also known to up-regulate β1 integrin expression in migrating keratinocytes[37]. Thus, the gating of charge in response to membrane depolarization by GPCRs might regulate both the function and expression of coassociating integrins. This raises the distinct possibility that the gating of charge by Kv channels might regulate the activity of coassociating integrins.

## INTEGRINS AS CRITICAL SENSORS OF DCEFS AND GALVANOTAXIS

We appreciate that many questions remain as to how dcEFs could regulate leukocyte migration and that it may be premature to speculate on how dcEFs are coupled to the molecular mechanics of cell migration. Nevertheless, it is our working hypothesis that when a cell experiences a physiological dcEF and undergoes migration towards the cathode, membrane depolarization at the leading edge is the primary stimulus for modifying integrin function and that integrins are the critical sensors of a dcEF. We speculate that the conformation of β1 integrins, in particular, is sensitive to small changes in membrane potential. This might arise indirectly through integrin coassociation with voltage-gated ion channels (or GPCRs) in which a conformational change within the voltage-sensor is relayed or transmitted to the integrin[3,4,40]. An alternative explanation is that integrins possess an intrinsic mechanism by which they are responsive to changes in membrane potential. This could occur, for example, if an electric dipole moment existed within a critical regulatory domain of β1 integrin that responded to changes in membrane potential. In proteins, the most likely source of a significant electric dipole moment that would interact with an electric field generated by a membrane potential would be an α-helix buried within the globular fold of a protein domain or lipid bilayer[41]. Importantly, when an electric dipole moment lies orthogonal to an electric field, it will experience a force, or torque, which may be sufficient to impose a significant conformational change.

This is precisely the mechanism by which voltage sensors function within voltage-gated ion channels where small changes in membrane potential can have dramatic effects on their gating properties. Thus, depolarization will also affect the activation, or open probability, of Kv channels, which should be sufficient to promote $K^+$ efflux and, as discussed above, integrin-dependent adhesion. Therefore, we view β1 integrin-dependent adhesion as being secondary to membrane potential changes and ion channel activity. This does not argue against those reports where integrin engagement was responsible for cellular hyperpolarization, which may be due to intracellular signaling events affecting surface expression or activity of ion channels.

## IMPLICATIONS FOR PECAM-1 IN LEUKOCYTE MIGRATION

Our primary interest in recent years has been the cell adhesion molecule PECAM-1, an important regulator of leukocyte transmigration across both the endothelium and perivascular basement membrane[42,43,44,45,46]. Of particular interest and relevance, PECAM-1 not only mediates the active detachment of live neutrophils from macrophages, but also influences the direction and velocity of migration of neutrophils across an endothelial surface, a distinct vector property[47,48]. This raises the possibility that PECAM-1 may be coupled to the ability of neutrophils to sense dcEFs. PECAM-1 is thought to function, in part, by modulating β1, β2, and β3 integrin activity[46]. The mechanism by which CD31 regulates integrin function is not known, although we recently reported that homophilic ligation or antibody cross-linking of PECAM-1 expressed by macrophages inhibited $K^+$ current through a specific voltage-gated potassium channel, Kv11.1[34]. Kv11.1 is known to associate physically with β1 integrins (Table 1, Part A ). Our experiments also showed that ERG was responsible for the rapid repolarization of the macrophage membrane potential following deliberate depolarization. By inhibiting ERG current, either pharmacologically or by the ligation of PECAM-1, we were able to promote macrophage β1 integrin binding of apoptotic cells or Latex beads that had been opsonized with fibronectin[34,49]. This observation is analogous to that observed by Levite et al. where deliberate depolarization of T-lymphocytes while inhibiting $K^+$ efflux via Kv1.3 was sufficient to activate β1 integrin function[35]. Thus, membrane depolarization was an initiating signal for integrin activation.

## CONCLUDING REMARKS

Ample evidence exists to confirm a physical and functional association between β1 integrins and Kv channels. Understanding how β1 integrins and Kv channels cross-talk should provide useful insights into integrin and cellular function. It is our suggestion, however, that integrins not only integrate intra- and extracellular molecular signaling events, but they also integrate electrical signals to modify cell behavior. It is implicit in our review that the cell adhesion molecule PECAM-1 functions to regulate cell adhesion and migration by regulating the electrical properties of cells, specifically the repolarization of a depolarized membrane potential where depolarization is an electrical signal for a change in integrin conformation.
